# Temperature Rises in the Pulp Chamber with Different Techniques of Orthodontic Adhesive Removal 

**DOI:** 10.22037/iej.v12i3.16635

**Published:** 2017

**Authors:** Maurício Barbieri Mezomo, Juliana Abreu, Juliana Weber, Renato Dalla Porta Garcia, José Antônio Poli Figueiredo, Eduardo Martinelli de Lima

**Affiliations:** a *Professor of Orthodontics, Faculty of Dentistry, Franciscan University Center, Santa Maria, RS, Brazil; *; b *Private Practice, Santa Maria, RS, Brazil;*; c *Post-graduate Student (PhD), Faculty of Dentistry, Lutheran University of Brazil, Canoas, RS, Brazil; *; d * Professor of Endodontics, Faculty of Dentistry, Pontifical Catholic University of Rio Grande do Sul, Porto Alegre, RS, Brazil; *; e * Professor of Orthodontics, Faculty of Dentistry, Pontifical Catholic University of Rio Grande do Sul, Porto Alegre, RS, Brazil*

**Keywords:** Enamel Clean-Up, Pulp Chamber, Pulp Temperature, Temperature Rise

## Abstract

**Introduction:**

The aim of this *in vitro* study was to compare the temperature rises in the pulp chamber and time spent with different techniques for orthodontic resin adhesive removal.

**Methods and Materials:**

Adhesive removal was performed in 20 extracted human maxillary second premolars with five techniques: high-speed tungsten carbide burs with water-cooling (BurH-cool) and without cooling (BurH), low-speed carbide burs (BurL), low-speed aluminum-oxide discs (DiscL), and low-speed fiberglass burs (BurFGL). Pulp chamber temperature was measured with a thermocouple probe and time spent was recorded with a digital stopwatch. Comparisons of temperature rise and time between the techniques were performed with Analysis of variance and Tukey’s Honestly test. Correlation between variables was investigated with Pearson’s correlation coefficient.

**Results:**

Temperature rise and time were statistically different between techniques and showed a positive correlation between them (*r*=0.826) (*P*<0.01). BurH-cool provoked the lowest temperature rise and BurFGL the highest (*P*<0.01). Temperature rises were higher with DiscL than with BurH and BurL (*P*<0.01), which showed no statistical differences between them (*P*>0.05). The fastest technique was BurH-cool followed by BurL, BurH, DiscL and BurFGL (*P*<0.01).

**Conclusion:**

BurH-cool, BurH and BurL are safe adhesive removal techniques, whereas DiscL and BurFGL may damage pulp tissues. Time spent on adhesive removal has direct effect on temperature rise in the pulp chamber.

## Introduction

Temperature rises in the pulp chamber caused by dental procedures can damage the pulp tissues [1-6]. Zach and Cohen [7] reported that a temperature rise of 5.5^º^C in the pulp chamber provoked a high incidence of pulpal necrosis in primates. Thermal stimulation affects both efferent and afferent neurons in the dental pulp, but this organ does not thermo regulate as the skin. Small temperature changes do not cause microcirculatory reaction. However, noxious temperatures induce an increase in dental pulp blood flow with the participation of C fibers [8]. Together with the loss of substance P in small nerve fibers, there is a reduction of neurogenic hyperemia and plasma extravasation [9].

Dental procedures for resin adhesive removal may induce excessive temperature rises in the pulp chamber. Choice of adhesive removal technique, after brackets debonding, relies on effective enamel cleaning with low temperature rise. Adequate adhesive removal techniques must prevent vascular damage to the pulp and preserve the enamel morphology [10-12]. Standard methods for removing resin adhesives from enamel surfaces are the use of high-speed or low-speed tungsten carbide burs. These burs provoke minimal temperature rises, thus preventing damages to the pulp tissues [13, 14]. Usually, low-speed carbide burs leave smoother and more polished enamel surfaces than the high-speed burs [15, 16]. The use of aluminum-oxide discs and fiberglass burs are alternative methods for removing resin adhesives. In recent studies, these low-speed devices showed a better preservation of the enamel morphology than the carbide burs [15, 17-19]. However, no studies were found on the effects of aluminum-oxide discs or fiberglass burs in the pulp chamber temperature.

Clinicians should concern about temperature rises caused by adhesives removal techniques. Indeed, noxious temperatures might cause irreversible damage to the pulp. Therefore, the aim of this *in vitro* study was to compare the temperature rises in the pulp chamber and time spent with five orthodontic adhesive removal techniques. The null hypothesis was that there is no difference among adhesive removal techniques, neither on temperature rise in the pulp chamber, nor on time spent throughout the procedures.

## Materials and Methods

The Research and Ethics Committee of the Franciscan University Center (UNIFRA) approved this *in vitro* study. Sample size was calculated to a power of 90% and a bilateral significance level of 0.05 (Statistical Solutions, LLC Systems, Cottage Grove, WI, USA). Sample estimation was 18 human teeth to detect differences of 1.0^º^C in temperature rises in the pulp chamber (4.27±1.28^º^C). Because of possible sample losses throughout the study, 20 extracted human maxillary second premolars with intact crowns from the collection of the UNIFRA Dental School were selected.

Before the experiment, teeth were cleaned and stored in saline. Premolar roots were embedded in PVC cylinders filled with self-cured acrylic resin, leaving the crowns exposed. A 2-mm diameter access cavity to the pulp chambers was drilled in the occlusal surface using a spherical diamond bur (FG 1016, KG Sorensen, Kotia, SP, Brazil), allowing the thermometer sensor insertion. Buccal surfaces of teeth were cleaned using low-speed rubber cups soaked in pumice slurry; which were replaced for every ten specimens. After rinsing, the teeth were dried with air-jet for 10 sec. Enamel surfaces were etched with a 37% phosphoric acid gel for 20 sec, rinsed well and dried. Metal brackets (3M-Abzil, São José do Rio Preto, SP, Brazil) were bonded with Transbond XT (3M-Unitek, Monrovia, CA, USA), following manufacturer instructions; excess adhesive was removed from brackets edges using a dental probe. Photo-polymerization of resin adhesive was performed with a conventional LED-curing unit (RaddiCal, SDI, Bayswater, Victoria, Australia). Light curing was applied for 10 sec on mesial and distal sides of brackets. Debonding of brackets was carried out squeezing the mesial and distal wings of the brackets with a how plier. Preferably, most part of resin adhesive was kept adhered to teeth. Inspection under naked eye required more than 80% of resin adhesive remaining on enamel surface. A thermocouple probe with 1.6 mm-diameter (HI 766, Hanna Instruments, Leighton Buzzard, Bedfordshire, UK) was inserted in the pulp chamber and positioned against its buccal wall. Pulp chamber was filled with a silicone compound (Implastec, Votorantin, SP, Brazil) to transfer heat to the thermocouple (HI-935002, Hanna Instruments, Leighton Buzzard, Bedfordshire, UK). 

**Figure 1:**
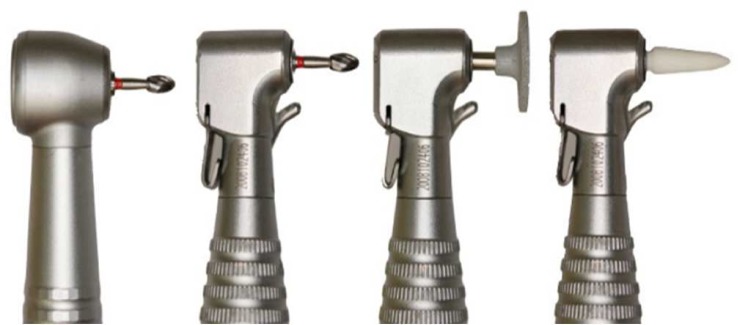
High-speed tungsten carbide bur (BurH; BurH-cool); low-speed tungsten carbide bur (BurL); low-speed aluminum-oxide disc (DiscL); and low-speed fiberglass bur (BurFGL

Five adhesive removal techniques were carried out in each premolar tooth (Table 1) (Figure 1). Following one technique, the bonding and debonding of brackets were repeated and subsequent techniques were applied in a random sequence. Procedure completion was determined by enamel surfaces free of resin adhesive at naked eye, under dental reflector light. BurH-cool (H379.314.014, Komet, Lemgo, Germany), BurH (H379.314.014, Komet, Lemgo, Germany), BurL (H379.314.014, Komet, Lemgo, Germany), DiscL (DU10CA, DHPro, Curitiba, PR, Brazil) and BurFGL (Fiberglass, TDV, Pomerode, SC, Brazil) were replaced after every 10 procedures. Temperature in the pulp chamber was measured throughout entire adhesive removal procedure. Differences between maximum and initial records on the thermometer display represented the temperature rise. Time elapsed between the beginning and completion of each procedure was measured with a digital stopwatch. The same operator performed resin adhesive removals using five techniques, with attention on minimizing eventual damage to the enamel surfaces.

***Statistical analysis***

Descriptive statistics were calculated for the temperature rise in the pulp chamber and time spent in each adhesive removal technique. Normal distribution of the data was ratified by the non-parametric Shapiro-Wilk test. Temperature rise and time were compared between techniques (BurH-cool, BurH, BurL, DiscL, BurFGL) using the Analysis of variance; Tukey’s Honestly post-hoc test was used to identify statistical significant differences. Analysis of correlation between temperature rise and time was performed using Pearson’s correlation coefficient (r). SPSS statistical software (SPSS version 20.0, IBM, Armonk, NY, USA) was used for analysis of the data. The level of significance was set at 0.05.

## Results

**Figure 2:**
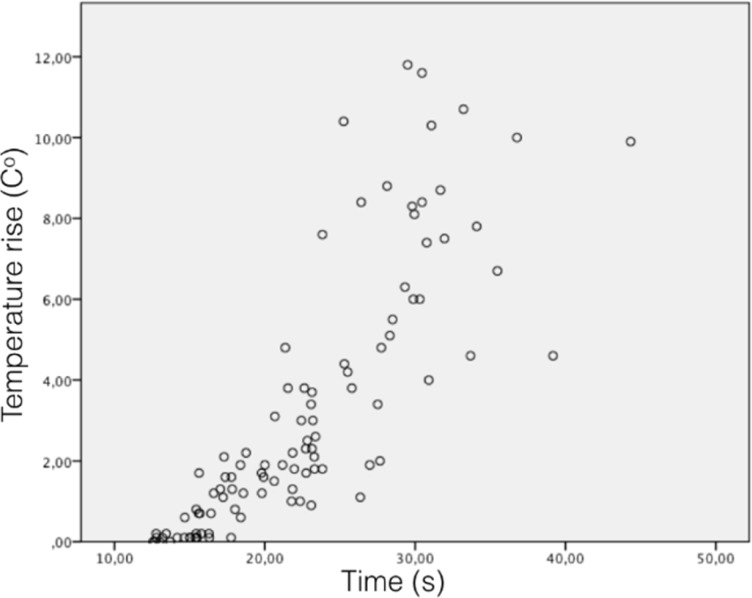
Correlation between the temperature rise and time spent

**Figure 3:**
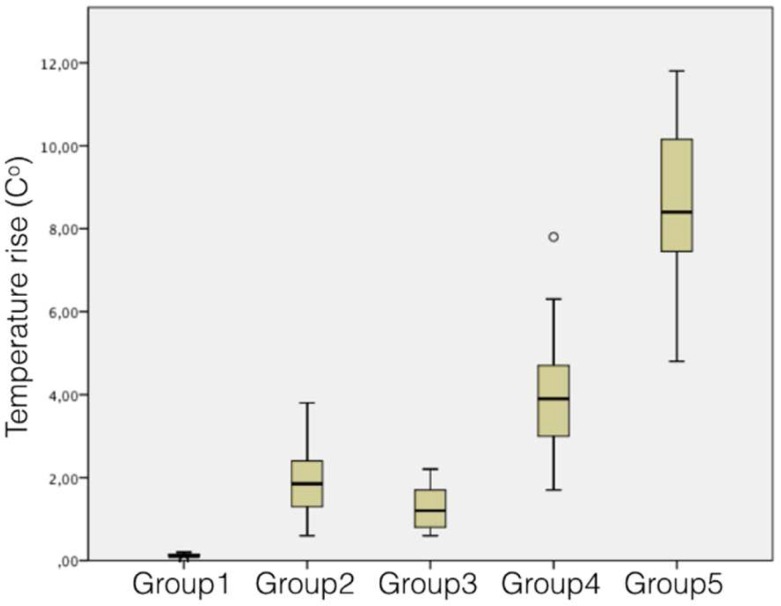
Pulp chamber temperature rises in a box-plot

Temperature rise and time spent showed statistically significant differences between resin adhesive removal techniques (*P*<0.01) (Table 2). Correlation between temperature rise and time spent was positive (*r*=0.826) and statistically significant (*P*<0.01) (Figure 2). BurH-cool (0.10±0.1^º^C) provoked the lowest temperature rise and BurFGL (8.57±1.9^º^C) the highest (*P*<0.01). Temperature rise with DiscL (3.94±1.6^º^C) was higher than with BurH (2.0±0.9^º^C) and BurL (1.28±0.5^º^C) (*P*<0.01), which showed no statistical differences between them (*P*>0.05) (Table 2). BurH-cool, BurH and BurL showed temperature rises below 4.8^º^C in all specimens. On the other hand, temperature rises were above 4.8^º^C in 25% of the specimens with DiscL and in 100% of the sample using BurFGL (Figure 3). The fastest technique was BurH-cool (15.6±1.5 sec), followed by BurL (18.5±2.7 sec), BurH (22.4±2.7 sec), DiscL (25.8±5.8 sec) and BurFGL (30.9±4.4 sec) (*P*<0.01) (Table 2).

## Discussion

This *in vitro* study compared the temperature rises in the pulp chamber and time spent with five adhesive removal techniques (Table 1), which were performed in 20 human maxillary second premolars. Teeth morphology, the enamel features and dentinal thickness were standardized as the same sample was used for adhesive removal with different techniques. Moreover, a clinical situation of brackets re-bonding was resembled [20]. Both the temperature rise and the time spent showed statistical significant differences between techniques (BurH-cool, BurH, BurL, DiscL, BurFGL). Thus, the null hypothesis was fully rejected.

Heat produced during resin adhesives removal procedures depends on the size type and abrasiveness of the instruments, the duration of contact, and amount of remaining adhesive [21, 22]. In the present study, temperature rise with BurH-cool was irrelevant. The use of BurH and BurL caused higher temperature rises in the pulp chamber (Table 2), but these rises were below 4.8^o^C in all samples (Figure 3). These results are in line with the findings of Uysal *et al.* [14]. Clinically, BurH and BurL (no water-cooling) allowed better distinguishing between the enamel and resin adhesive limits. Bicakci *et al.* [23] carried out a histopathologic evaluation in premolars extracted for orthodontic purposes and reported reversible alterations in the pulp tissues, after resin adhesive removal with tungsten carbide burs without cooling.

**Table 1:** Techniques for orthodontic resin adhesive removal

	
**BurH-cool**	high-speed 12-blade tungsten carbide bur with water-cooling (Lemgo, Germany)
**BurH**	high-speed 12-blade tungsten carbide bur without water-cooling (Lemgo, Germany)
**BurL**	low-speed 12-blade tungsten carbide bur (Lemgo, Germany)
**DiscL**	low-speed aluminum-oxide disc (DU10CA, Dhpro, Paranaguá, PR, Brazil)
**BurFGL**	low-speed fiberglass bur (3102, TDV, Pomerode, SC, Brazil)

**Table 2:** Descriptive statistics: temperature rise in the pulp chamber and time spent in the technique, interaction between techniques

**Technique**	**Temperature rise**			**Time spent**		
	**Mean (SD)**	**Min-Max**	**Sig**	**Mean (SD)**	**Min-Max**	**Sig**
**BurH-cool**	0.10 (0.1)	0.0-0.2	A*	15.6 (1.5)	12.6-17.8	A**
**BurH**	2.0 (0.9)	0.6-3.8	B**	22.4 (2.7)	17.4-27.7	C*
**BurL**	1.28 (0.5)	0.6-2.2	B*	18.5 (2.7)	14.7-23.8	B**
**DiscL**	3.94 (1.6)	1.7-7.8	C**	25.8 (5.8)	15.6-39.2	D*
**BurFGL**	8.57 (1.9)	4.8-11.8	D**	30.9 (4.40	23.8-44.3	E**

Analysis of variance; Tukey-Honestly post-hoc test (P<0.05). Different letters indicate statistical differences

SD indicates standard deviation; Min, minimum; Max, maximum; Sig, significance; °C, Celsius degrees; s, seconds; BurH-cool, high-speed carbide bur with cooling; BurH, high-speed carbide bur without cooling; BurL, low-speed carbide bur; DiscL, low-speed aluminum oxide disc; BurFGL, low-speed fiberglass bur

*= P<0.05;

** =P<0.01).

Recent studies [15, 17-19] reported less enamel scars and more polished enamel surfaces when using aluminum-oxide discs and fiberglass burs for resin adhesive removal. Ryf *et al.* [24] found an average enamel loss of 4.1 µm after adhesive removal with tungsten carbide burs. When abrasive discs were associated to burs, enamel loss was reduced to 2.9 µm. Combination of techniques was useful on preserving dental enamel during resin adhesive removal. In the current study, DiscL caused temperature rises in the pulp chamber above 4.8^º^C in 25% of the sample (Figure 3). Maximum temperature rise was 7.8^º^C (Table 2), being potentially harmful to the pulp tissues. Nevertheless, DiscL allowed an optimal distinguish between enamel and resin adhesive. One could suggest intermittent use of DiscL with short time intervals, in order to avoid excessive temperature rise during resin adhesive removal. 

The use of BurFGL for resin adhesive removal provoked the greatest temperature rise in the pulp chamber (8.5±1.9^º^C) (Table 2). Temperature rise with BurFGL was above 4.8^º^C in 100% of the sample (Figure 3). This outcome revealed that the use of BurFGL for resin adhesive removal is highly dangerous to the pulp tissues. Clinician must be aware of using BurFGL for resin adhesive removal in sound teeth. Time spent during resin adhesive removal had a high correlation with temperature rise in the pulp chamber (*r*=0.826). Procedure time determined 68% of the temperature rise. BurFGL demanded up to 44 sec to be completed and showed 11.8^º^C of maximum temperature rise. The greater was the time spent during adhesive removal, the greater was the temperature rise in the pulp chamber. Temperature rises and time spent followed the same sequence of increase among the adhesive removal techniques (Table 2). 

This *in vitro* study has clear limitations. In *in vivo *conditions, the blood circulation into the pulp chamber and the fluid movement into the dentinal tubules interfere in the heat conduction inside the tooth and can produce a different temperature response to the resin adhesive removal process [24]. In addition, the surrounding periodontal tissues promote the dispersion of heat, limiting the increase in the pulp temperature. The temperature rise might be higher in young teeth due to the greater volume of the pulp and to the thinner thickness of the dentin. In older teeth, the deposition of secondary dentin is enhanced. 

Outcomes of the present study enrich the current knowledge on temperature rises in the pulp chamber caused by dental procedures for resin adhesive removal. The use of BurH-cool, BurH and BurL spent a shorter time and produced low temperature rises. Differently, DiscL and BurFGL lasted longer during adhesive removal and caused temperature rises above 5.5^º^C, especially the latter. In addition to clinician preference, choice of resin adhesive removal techniques must rely on effective enamel cleaning with low temperature rise. Alternative procedures could be thought towards adhesive removal, such as ultrasonic with abundant water-cooling. However, if used in dry mode, at least for removal of metallic posts, injurious heat transfer occurs in less than one min [25].

## Conclusion

BurH-cool, BurH and BurL adhesive removal techniques are safe for the pulp. However, DiscL and BurFGL might be dangerous for the pulp tissues. Time spent during adhesive removal has direct effect on temperature rise in the pulp chamber.
